# The Sensitivity of Endodontic *Enterococcus* spp. Strains to Geranium Essential Oil

**DOI:** 10.3390/molecules201219888

**Published:** 2015-12-21

**Authors:** Monika E. Łysakowska, Monika Sienkiewicz, Katarzyna Banaszek, Jerzy Sokołowski

**Affiliations:** 1 Department of Microbiology and Medical Laboratory Immunology, Medical University of Lodz, 92-231 Lodz, Pomorska 251, Poland; 2 Department of Environmental Biology, Medical University of Lodz, 90-752 Lodz, Żeligowskiego 7/9, Poland; monika.sienkiewicz@umed.lodz.pl; 3 Department of General Dentistry, Medical University of Lodz, 92-231 Lodz, Pomorska 251, Poland; katarzyna.banaszek@umed.lodz.pl (K.B.); jerzy.sokolowski@umed.lodz.pl (J.S.)

**Keywords:** enterococci, endodontic treatment, geranium essential oil

## Abstract

Enterococci are able to survive endodontic procedures and contribute to the failure of endodontic therapy. Thus, it is essential to identify novel ways of eradicating them from infected root canals. One such approach may be the use of antimicrobials such as plant essential oils. Enterococcal strains were isolated from endodontically treated teeth by standard microbiological methods. Susceptibility to antibiotics was evaluated by the disc-diffusion method. The minimal inhibitory concentration (MIC) of geranium essential oil was investigated by microdilution in 96-well microplates in Mueller Hinton Broth II. Biofilm eradication concentrations were checked in dentin tests. Geranium essential oil inhibited enterococcal strains at concentrations ranging from 1.8–4.5 mg/mL. No correlation was shown between resistance to antibiotics and the MICs of the test antimicrobials. The MICs of the test oil were lower than those found to show cytotoxic effects on the HMEC-1 cell line. Geranium essential oil eradicated enterococcal biofilm at concentrations of 150 mg/mL. Geranium essential oil inhibits the growth of endodontic enterococcal species at lower concentrations than those required to reach IC_50_ against the HMEC-1 cell line, and is effective against bacteria protected in biofilm at higher concentrations. In addition, bacteria do not develop resistance to essential oils. Hence, geranium essential oil represents a possible alternative to other antimicrobials during endodontic procedures.

## 1. Introduction

Enterococcal strains are frequently found in the human digestive and genitourinary tracts. Even though enterococci may be minor inhabitants of the oral cavity of healthy individuals [1], they are frequently isolated in persistent chronic endodontic failures [2]. Therefore, the eradication of enterococci from infected canals is crucial for the preservation of treated teeth loss. The removal of enterococci is also hindered by their resistance to some groups of antibiotics [3]. If resistant, they are able to exchange their resistance genes with the other species [4]. Consequently, it is necessary to identify their sensitivity to antibiotics, which, firstly, may assist in planning the most appropriate therapy and, secondly, may prevent the spread of resistance to other species.

Therefore, it is highly important to identify novel ways of eradicating these pathogens from infected root canals. One way of preventing bacterial infections could be by the use of essential oils, as these possess many valuable properties, bestowing antimicrobial, anti-inflammatory and antioxidant effects [5], they can be used to treat infections of the respiratory, digestive and genitourinary systems, as well as skin disorders, and can be used as natural preservatives in cosmetics, or drugs [6]. Some essential oils have been tested in dentistry. Rao *et al.* recommended the use of essential oils as additives to antibacterial agents against oral pathogens, and to food products and mouthwashes for preventing and treating dental caries. They investigated the activity of essential oils against some oral microorganisms such as *Streptococcus mutans*, *Streptococcus salivarius*, *Streptococcus mitis*, *Lactobacillus fermentum*, *Streptococcus anginosus*, *Streptococcus mutans*, *Streptococcus gordonii*, *Lactobacillus acidophilus* and *Staphylococcus aureus* obtaining good results [7].

Geranium oil from *Pelargonium graveolens* A is known to have many valuable properties in anti-infective therapy. Geranium oil has been shown to have an antibacterial effect against reference strains of *Escherichia coli* ATCC 25922, *Klebsiella pneumoniae* ATCC 15380, *Pseudomonas aeruginosa* ATCC 27853, and *Staphylococcus aureus* ATCC 25923 [8]. The cutaneous application of this oil may suppress the inflammatory symptoms related to neutrophil accumulation and edema [9] and it has been shown to have a synergistic effect against *Staphylococcus aureus* when combined with norfloxacin [10].

To continue this line of investigation, we decided to determine whether *Enterococcus* spp. isolated from endodontic infections may also be inhibited by geranium essential oil. Thus, the aims of the study were to evaluate the susceptibility to geranium essential oil of enterococcal strains in: (a) planktonic state and (b) grown on dentin.

## 2. Results

### 2.1. Bacterial Strains and Their Susceptibility to Antibiotics

The test strains comprised 21 *Enterococcus* spp. endodontic strains showing diverse resistance to antibiotics. Detailed results for each endodontic enterococcal strain are shown in Table 1. None of the *Enterococcus* strains were resistant to penicillins, gentamicin, linezolid or teicoplanin. 

**Table 1:** Susceptibility pattern of *Enterococcus* strains.

No.	Species	Susceptibility to Antibiotics											
		AMP	C	CIP	E	GM	LNZ	P	S	TE	TEC	TGC	VA
1.	*E. faecalis* (d1Efs)	S	S	R	I	S	S	S	S	S	S	R	S
2.	*E. faecalis* (d2Efs)	S	R	S	I	S	S	S	S	R	S	R	S
3.	*E. faecalis* (d4Efs)	S	S	S	I	S	S	S	S	S	S	R	S
4.	*E. faecalis* (d5Efs)	S	S	S	I	S	S	S	S	S	S	R	S
5.	*E. faecalis* (d6Efs)	S	S	I	S	S	S	S	S	S	S	S	S
6.	*E. faecalis* (d7Efs)	S	S	S	S	S	S	S	S	R	S	S	S
7.	*E. faecalis* (d8Efs)	S	S	I	I	S	S	S	S	S	S	R	S
8.	*E. faecalis* (d9Efs)	S	S	S	I	S	S	S	S	S	S	R	S
9.	*E. faecalis* (d10Efs)	S	I	I	I	S	S	S	S	R	S	R	S
10.	*E. faecalis* (d11Efs)	S	S	I	I	S	S	S	S	S	S	S	S
11.	*E. faecalis* (d13Efs)	S	S	S	S	S	S	S	S	S	S	S	S
12.	*E. faecalis* (d15Efs)	S	S	S	R	S	S	S	S	R	S	S	S
13.	*E. faecalis* (d16Efs)	S	S	I	S	S	S	S	S	S	S	S	S
14.	*E. faecalis* (d17Efs)	S	S	I	S	S	S	S	S	R	S	S	S
15.	*E. faecalis* (d18Efs)	S	S	I	I	S	S	S	S	S	S	S	S
16.	*E. faecalis* (d19aEfs)	S	S	S	R	R	S	S	S	S	S	S	S
17.	*E. casseliflavus* (d19bEcas)	S	S	S	R	R	S	S	S	S	S	S	R
18.	*E. faecalis* (d20Efs)	S	S	S	S	S	S	S	S	S	S	S	S
19.	*E. faecalis* (d21Efs)	S	S	S	S	S	S	S	S	S	S	S	S
20.	*E. faecalis* (d22Efs)	S	S	S	I	S	S	S	S	R	S	S	S
21.	*E. faecalis* (d24Efs)	S	S	S	R	S	S	S	R	R	S	S	S

AM: ampicillin, C: chloramphenicol, CIP: ciprofloxacin, E: erytromycin, GM: gentamycin, LNZ: linezolid, P: penicillin, S: streptomycin, TE: tetracycline, TEC: teicoplanine, TIG: tigecycline, VA: wancomycin; S: sensitive strain, I: intermediate sensitive strain, R: resistant strain.

One *E. faecalis* strain presented an HLSR phenotype (high level resistance to streptomycin) while the *E. casseliflavus* strain was resistant to vancomycin. Eight strains were resistant (intermediately resistant or resistant) to ciprofloxacin (38.1%), fourteen were resistant to erythromycin (66.7%), seven to tetracycline and tigecycline (33.3%).

### 2.2. Constituents of the Test Oil

Sixty-one constituents were identified in the test geranium oil, as described previously [11]. Briefly, citronellol (26.7%) and geraniol (13.4%) were present in the most significant amounts. The other major components of the geranium oil were nerol (8.7%), citronellyl formate (7.1%), isomenthone (6.3%), linalool (5.2%), 10-*epi*-γ-eudesmol (4.4%), geranyl formate (2.5%), menthone (1.6%), β-caryophyllene (1.5%), geranyl butyrate (1.4%), *cis*-rose oxide (1.4%), geranial (1.1%), and β-baurobonene (1.1%). Other components were below 1% [11]. The composition of the test essential oil was consistent with the ISO-4731 standard. The analysis of the test essential oil showed that its composition meets the requirements of the Polish Pharmacopoeia IX [12] and the European Pharmacopoeia 8 [13].

### 2.3. Susceptibility of Enterococcal Strains to Geranium Essential Oil

Generally, endodontic enterococcal strains were inhibited by lower concentrations of geranium essential oil when compared to other results [8,14]. Sixteen strains (76.2%) were inhibited by concentrations in the 1.8–3.15 mg/mL range and only five (23.8%) by concentrations ranging from 3.6–4.5 mg/mL. No differences were noticed between two MIC groups of chlorhexidine (3 and 6 μg/mL) and MICs of the test essential oil (*p* = 1.0). Similarly, no difference was found between the MICs of geranium essential oil against antibiotic-resistant strains or antibiotic-sensitive strains (*p* = 0.603). Table 2 presents MIC values for geranium and chlorhexidine.

**Table 2:** Susceptibility of *Enterococcus* strains to geranium oil and chlorhexidine.

Enterococcus Strain No.	Geranium Oil (MIC)/mg/mL	Chlorhexidine (MIC)/mg/mL
1. *E. faecalis* (d1Efs)	4.5	0.006
2. *E. faecalis* (d2Efs)	2.25	0.006
3. *E. faecalis* (d4Efs)	2.25	0.006
4. *E. faecalis* (d5Efs)	4.5	0.006
5. *E. faecalis* (d6Efs)	4.5	0.003
6. *E. faecalis* (d7Efs)	3.15	0.003
7. *E. faecalis* (d8Efs)	3.15	0.003
8. *E. faecalis* (d9Efs)	2.25	0.003
9. *E. faecalis* (d10Efs)	3.15	0.006
10. *E. faecalis* (d11Efs)	3.15	0.006
11. *E. faecalis* (d13Efs)	2.25	0.006
12. *E. faecalis* (d15Efs)	2.25	0.003
13. *E. faecalis* (d16Efs)	3.15	0.006
14. *E. faecalis* (d17Efs)	2.25	0.006
15. *E. faecalis* (d18Efs)	2.25	0.006
16. *E. faecalis* (d19aEfs)	4.5	0.003
17. *E. casseliflavus* (d19bEcas)	2.25	0.006
18. *E. faecalis* (d20Efs)	2.25	0.006
19. *E. faecalis* (d21Efs)	3.6	0.006
20. *E. faecalis* (d22Efs)	1.8	0.003
21. *E. faecalis* (d24Efs)	2.25	0.003
22. *E. faecalis* 29212 ATCC	2.25	0.003
23. *E. faecalis* 51299 ATCC	2.25	0.003

### 2.4. The Effect of Geranium Essential Oil on the Viability of Human Microvascular Endothelial Cells (HMEC-1)

In general, a dose-dependent decrease in the survival of the cell line was observed. The geranium oil exhibited cytotoxicity against HMEC-1 with IC_50_ = 8.25 μL/mL (7.42 mg/mL) [15].

### 2.5. Efficacy of Geranium Essential Oil on MICROORGANISMS in Dentin

No bacterial growth was observed in negative controls. The detailed results of the antimicrobial activity tests are shown in Table 3. The 5 mg/mL and 25 mg/mL concentrations showed low killing efficacy. Higher concentrations were more effective at killing enterococci, but a concentration of 150 mg/mL was required to kill all microorganisms.

**Table 3:** Antimicrobial activity of geranium essential oil against *E. faecalis* biofilm in dentin.

Dose	Antimicrobial Activity against *E. faecalis* Biofilm	
	Units without Biofilm Eradication	Kill Percentage Mean
5 mg/mL	6/6	98.70%
25 mg/mL	6/6	99.50%
50 mg/mL	6/6	99.96%
75 mg/mL	6/6	99.97%
100 mg/mL	5/6	99.97%
125 mg/mL	1/6	99.99%
150 mg/mL	0/6	100%

## 3. Discussion

*E. faecalis* strains may present resistance to antibiotics and express a diverse range of virulence factors which may lead to endodontic treatment failures [16]. Therefore, studies concerning the antimicrobial efficacy of new antimicrobial products are of great importance. One possible solution is the use of natural products such as essential oils. Some extracts from Brazilian forest plants showed significant antimicrobial effects against standard *E. faecalis* ATCC 29212 strain, e.g., an extract from *Ipomoea alba* L. (*Convolvulaceae*) presented a Minimal Bactericidal Concentration (MBC) of ≤0.04 mg/mL [17]. However, there is shortage of data concerning the activity of such essential oils against endodontic isolates and their cytotoxicity.

The beneficial antibacterial effect of *Pelargonium graveolens* A. essential oil investigated in this *in* *vitro* study is attributed mainly to the presence of geraniol [18]. Others have noted that the activity of essential oils is correlated with the presence of terpenoid phenols (carvacrol, eugenol, thymol) or some oxygenated monoterpenes (nerol, linealool, α-terpineol, fenchol, terpinen-4-ol [6,19,20]. The tested geranium essential oil contains mainly citronellol (26.7%), geraniol (13.4%) and nerol (8.7%), which show similar antibacterial activity.

The previous findings indicate that geranium oil may play a role in treating dermatological problems, as it is known to inhibit the clinical multidrug resistant Gram-negative bacteria isolated from difficult-to-heal wounds [11]. Good results were observed for Gram-positive bacteria where the MIC of geranium EO for *S. aureus* was >12.8 mg/mL, and for *B. subtilis* it was >6.4 mg/mL [8]. Malik *et al.* [12] showed that MIC of geranium EO for *S. aureus* was 8.98 mg/mL. The results of the present study show that test oil had MIC values ranging from 1.8–4.5 mg/mL when used against clinical strains of *E. faecalis* isolated from root canals. The same geranium oil was less active against *A. baumannii* strains isolated from clinical materials, hospital equipment and the environment (MICs = 7.5 and 8.0 μL/mL). Such differences may be attributed to the presence of cell wall lipopolysaccharides in Gram-negative bacteria, which decrease the rate of diffusion [21].

Zore *et al.* reported that 561 μg/mL^−1^ geraniol was non-toxic in HeLa cells at MICs for *C. albicans* growth [18]. Our recent results confirmed that geranium essential oil is effective against enterococci at concentrations lower than those at which toxicity to human HMEC-1 cells had formerly been observed (at IC_50_ = 8.25 μL/mL). These results show that geranium oil may be safer towards HMEC-1 cells than the other previously tested oils [15] and may be an alternative antimicrobial to use in endodontic procedures. A current literature review indicates this to be the first study on the activity of *Pelargonium* essential oil against clinical *E. faecalis* strains isolated from root canal infections. The results are promising, especially bearing in mind that bacteria are not able to develop easily resistance to essential oils because of their complex nature. Multiple oil compounds may disturb several targets in the bacterial cell at the same time, *i.e.*, the cell wall and membranes, ATP production, protein synthesis, pH, and DNA [22]. Geranium essential oil, despite acting on cell wall membranes, is known to be a very effective quorum-sensing inhibitor [23]. When the susceptibility to antibiotics was compared with that of geranium essential oil, it became evident that no correlation existed between antibiotic resistance and MIC value of the essential oil (*p* = 0.603). The strains which were inhibited by higher concentrations of geranium essential oil presented diverse resistance profiles.

The activity of test geranium oil on enterococcal biofilm was also interesting. Previously, a difference in antimicrobial activity was observed for geranium essential oil against resistant and sensitive strains in a biofilm state, which might suggest better biofilm synthesis among resistant isolates [24]. Benbelaid *et al.* [19] concluded that multidrug resistant enterococci were more resistant to essential oils mainly due to the ability to produce biofilm. In our study, the antimicrobial activity of geranium essential oil against a 3-week biofilm was assayed in dentin. Such well-established, bacterial communities are often observed in persistent infections and are difficult to eradicate, and it was found that a concentration of 125 mg/mL of geranium essential oil was required to remove the majority of bacteria and 150 mg/mL to eradicate 3-week *E. faecalis* biofilm. Those concentrations are thirty times higher than the MICs of the test oil. However, the advantage of this natural oil over simple chemicals (e.g., chlorhexidine) is that microorganisms have not developed resistance to it yet [25,26,27], probably because of the presence of multiple compounds in the oil, which may show diverse mechanisms of action. Because of the fact that geranium essential oil showed noteworthy antibacterial activity future *in vivo* studies of its activity are planned.

## 4. Experimental Section

### 4.1. Bacterial Strains

Enterococcal strains (*n* = 21) were isolated from endodontically treated teeth in the Department of General Dentistry, Medical University of Lodz, Poland. The sampling procedure was described previously [28]. Briefly, microbial samples were obtained directly from the root canal of the involved tooth, after cleaning the teeth with pumice and water, removing caries, and isolation of teeth from the oral cavity with a previously disinfected rubber dam (Hygenic Dental Dam Kit, Coltene/Whaledent, Altstätt, Switzerland). To provide aseptic conditions, the field was disinfected with 30% hydrogen peroxide and 5.25% sodium hypochloride and then inactivated by 5% sodium thiosulfate (Avantor Performance Materials Poland S.A., Gliwice, Poland). The sterility of the operative field and access cavity was verified by taking a swab sample (Graso, Jabłowo, Poland). Two sterile paper points (Aceone Dent Korea Industrial Company, Bucheon, South Korea) were introduced to the predetermined working length and held in place for 1 minute to absorb the canal contents. Afterwards, the tested and control paper points (sterilized before use), without any contact with the investigated tooth, were immediately transferred to 3 mL of RTF medium [29].

Enterococci were cultured according to standard microbiological methods. Identification to species level was performed with API20 Strep (bioMerieux, Marcy l’Etoile, France), and then confirmed by PCR for d-alanine-d-alanyl ligase (*ddl*). The PCR profile and detection of products was as previously described [30]. Susceptibility to antibiotics was evaluated by the disc-diffusion method on Mueller-Hinton II Agar (bioMerieux) for the following antibiotics: penicillin (10 U), ampicillin (10 μg), erythromycin (15 μg), ciprofloxacin (5 μg), gentamicin (120 μg), streptomycin (300 μg), chloramphenicol (30 μg), linezolid (30 μg), tetracycline (30 μg), teicoplanin (30 μg), vancomycin (30 μg), and tigecycline (30 μg) (Becton Dickinson, Franklin Lakes, NJ, USA). The antibiotic discs were placed on Muller-Hinton agar seeded with 0.5 McFarland of test endodontic strains and incubated for 18 h (24 h for vancomycin). Inhibition zones were measured and the results were interpreted according to EUCAST [31] and Clinical and Laboratory Standards Institute guidelines [32]. The isolates were kept frozen at −70 °C in microbanks (Technical Service Consultants Ltd., Heywood, UK) until tested, as were the standard bacterial strains *Enterococcus faecalis* ATCC 29212 and *E. faecalis* ATCC 51299.

### 4.2. Analysis of Geranium Essential Oil

A commercial essential oils from *Pelargonium graveolens* A. was purchased from the manufacturer (POLLENA-AROMA, Warsaw, Poland) and analyzed by GC-FID-MS in the Institute of General Food Chemistry, Lodz University of Technology [11] using a Trace GC Ultra apparatus (Thermo Electron Corporation, Waltham, MA, USA) MS DSQ II detectors and FID-MS splitter (SGE). Identification of components was based on the comparison of their MS spectra with those of the laboratory-made MS library, commercial libraries (NIST 98.1, Wiley Registry of Mass Spectral Data, 8th Ed. and MassFinder 3.1) and with literature data [33,34] along with the retention indices on apolar column (Rtx-1, MassFinder 3.1) associated with a series of alkanes with linear interpolation (C_8_–C_26_).

### 4.3. Minimal Inhibitory Concentration (MIC) Assay

The Minimal Inhibitory Concentration (MIC) was determined by micro-dilution with the use of a 96-well microtiter plates. The geranium oil was diluted in ethanol. The stock solution was mixed with Mueller-Hinton broth to obtain concentrations from 1.0 mg/mL to 5.0 mg/mL for geranium oil. A 10 μL sample of inoculum containing 1.5 × 10^8^ CFU was added to 190 μL broth with various oil concentrations. In addition, a positive control consisting of broth without essential oil and a negative control consisting of broth without bacteria were prepared. The MIC values were evaluated after 24 h of incubation at 37 °C under aerobic conditions. The MIC endpoints were measured as the lowest concentration of antimicrobials at which there was no visible growth [35] and at A600 with a microplate reader (Multiscan Go; Thermo Scientific, Waltham, MA, USA). The control media containing only ethanol at concentrations used in the dilutions of tested oil did not inhibit the growth of bacteria.

### 4.4. Minimal Inhibitory Concentration (MIC) Assay—Chlorhexidine

The MICs of chlorhexidine (Amara, Cracow, Poland) were determined by micro-dilution with the use of a 96-well microtiter plates in Muller–Hinton broth (bioMerieux). Test and standard strains were investigated in three independent tests. Each isolate was tested in three repeats each time. The plates were incubated in 35 °C for 24 h. The test concentration of chlorhexidine ranged from 25 μg/mL to 0.78 μg/mL.

### 4.5. The Effects of Essential Oils on the Viability of Human Microvascular Endothelial Cells (HMEC-1)

Cell culture was purchased from ATCC and cultured as described previously [15]. For experimentation, cells between passages 10–31 were used. The viability of the HMEC-1 cells was measured using the 3-(4,5-dimethylthazol-2-yl)-2,5-diphenyltetrazolium bromide (MTT; Sigma-Aldrich Chemical Co. Ltd., Saint Louis, MO, USA) conversion method [15].

### 4.6. Antimicrobial Activity of Geranium Essential Oil on E. faecalis Biofilm in Dentin

#### 4.6.1. Bacteria Strain and Test Essential Oil

*E. faecalis* ATCC 29212 was used in this study. It was taken from overnight culture on 5% blood agar (Graso, Jabłonowo, Poland) at 37 °C. A test solution of bacteria was obtained as it was described previously [36]. The final test oil concentrations ranged from 5 to 150 mg/mL.

#### 4.6.2. The Dentin Blocks

The dentin blocks were prepared according to Baca *et al.* [36]. Twenty non-carious, freshly-extracted third human molars were sectioned using a Presi Mecatome T-201 system (Brie-et-Angonnes, France) at a high force of 5000 rpm with water cooling, at a cutting speed of 6 mm/min. Only a flat coronal dentin surface without enamel was used for the study. This slice was cut into serial blocks using a calibrator and polished with papers to obtain 2 × 2 × 2 mm (width × length × height) specimens. The dentin blocks were then submerged in 17% EDTA for 2 min followed by 2.5% NaOCl for 1 min to remove the smear layer formed during preparation, and finally sterilized. The sterile dentin blocks were then kept in sterile saline solution until used.

#### 4.6.3. Antimicrobial Activity

The test was performed according to Baca *et al.* [36] with some modifications. The wells of a 96-well microtiter plate were inoculated with 180 μL of the initial bacterial suspension, with two rows (16 wells) inoculated with sterile TSB to act as a sterility control. The sterile dentin blocks were submerged in the inoculated wells, and incubated for 3 weeks at 37 °C with 95% relative humidity. The TSB was refreshed every two days to ensure the growth of bacteria. After three weeks, the dentin cultures were rinsed twice with 180 μL of 0.9% sterile saline solution for 2 min.

The antimicrobial activity assay was performed in a microtiter plate with 100 mL of the test essential oil solutions per well. The last two wells in rows containing TSB acted as the sterility (dentin blocks without bacteria) and growth controls. The rinsed dentin blocks were then added to the test oil concentrations for 24 h. Following this, sterile absorbent paper disks (Oxoid, Hamphire, UK) were used to remove excessive liquid from the dentin blocks. These were placed in Eppendorf tubes with 200 μL TSB (Graso) and vortexed for 10 s. The biofilm was then recovered by sonication for 10 min. The obtained solutions were diluted serially from 10^−1^–10^−6^ in 0.9% saline, and 10 μL samples of these aliquots were plated on TSA for viable cell counting. Six replicates per essential oil concentration were performed. The antimicrobial activities of different concentrations of geranium essential oil were calculated for each group of dentin blocks (for different geranium essential oil concentrations) as follows: (1 – (mean CFU_GEO_/mean CFU control)) × 100% [36].

### 4.7. Statistical Analysis

To determine the correlation between antibiotic resistance and MICs to geranium essential oil, the antibiotic resistance values were categorized. The first group tested consisted of sensitive strains (sensitive or intermediately sensitive) while the second group consisted of resistant strains (resistant to at least one of test antimicrobials). The following results were compared: a) sensitivity to antibiotics to MICs of geranium essential oil, and b) chlorhexidine MICs (1st group—0.003 mg; 2nd—0.006 mg) and MICs of essential oil. Before further analysis, the results were tested for normality using the Shapiro-Wilk test. As the results were significantly non-normal, the Mann Whitney test was used to analyze the data. A *p*-value of 0.05 was considered statistically significant in this study.

## 5. Conclusions

An inhibitory effect of geranium essential oil was shown against endodontic enterococcal strains sensitive to antibiotics as well as these which were drug resistant. Therefore, the use of this oil may represent an alternative strategy for avoiding persistent endodontic infections. However, the concentration of essential oil is an important issue which demands further study, as at higher concentrations, toxicity to human cells has been observed.

## References

[1] Sedgley C.M., Lennan S.L., Clewell D.B. (2004). Prevalence, phenotype and genotype of oral enterococci. Oral Microbiol. Immunol..

[2] Sedgley C.M., Molander A., Flannagan S.E., Nagel A.C., Appelbe O.K., Clewell D.B., Dahlén G. (2005). Virulence, phenotype and genotype characteristics of endodontic *Enterococcus* spp.. Oral Microbiol. Immunol..

[3] Hollenbeck B.L., Rice L.B. (2012). Intrinsic and acquired resistance mechanisms in *Enterococcus*. Virulence.

[4] De Niederhäusern S., Bondi M., Messi P., Iseppi R., Sabia C., Manicardi G., Anacarso I. (2011). Vancomycin-resistance transferability from VanA enterococci to *Staphylococcus aureus*. Curr. Microbiol..

[5] Reichling J., Schnitzler P., Suschke U. (2009). Essential oils of aromatic plants with antibacterial, antifungal, antiviral and cytotoxic properties—An oview. Forsch. Komplementmed..

[6] Edris A.E. (2007). Pharmaceutical and therapeutic potentials of essential oils and their individual volatile constituents: A review. Phytother. Res..

[7] Rao P.K., Bobbarala V., Aryamithra D., Devi P.S., Rao T.R. (2008). *In vitro* antibacterial activities of plant essential oils against oral bacteria. Ind. J. Multidiscip. Res..

[8] Prabuseenivasan S., Jayakumar M., Ignacimuthu S. (2006). *In vitro* antibacterial activity of some plant essential oils. BMC Complement. Altern. Med..

[9] Maruyama N., Sekimoto Y., Ishibashi H., Shigeharu I., Oshima H., Yamaguchi H., Abe S. (2005). Suppression of neutrophil accumulation in mice by cutaneous application of geranium essential oil. J. Inflamm..

[10] Rosato A., Vitali C., de Laurentiis N., Armenise D., Milillo M.A. (2007). Antibacterial effect of some essential oils administered alone or in combination with Norfloxacin. Phytomedicine.

[11] Sienkiewicz M., Poznańska-Kurowska K., Kaszuba A., Kowalczyk E. (2013). The antibacterial activity of geranium oil against Gram-negative bacteria isolated from difficult-to-heal wounds. Burns.

[12] (2011). Polish Pharmacopeia.

[13] (2013). European Pharmacopoeia.

[14] Malik T., Singh P. (2010). Antimicrobial effects of essential oils against uropathogens with varying sensitivity to antibiotics. Asian J. Biol. Sci..

[15] Sienkiewicz M., Głowacka A., Kowalczyk E., Wiktorowska-Owczarek A., Jóźwiak-Bębenista M., Łysakowska M. (2014). The biological activities of cinnamon, geranium and lavender essential oils. Molecules.

[16] Lin L.M., Skribner J.E., Gaengler P. (1992). Factors associated with endodontic treatment failures. J. Endod..

[17] Castilho A.L., Saraceni C.H., Díaz I.E., Paciencia M.L., Suffredini I.B. (2013). New trends in dentistry: Plant extracts against *Enterococcus faecalis*. The efficacy compared to chlorhexidine. Braz. Oral Res..

[18] Zore G.B., Thakre A.D., Rathod V., Karuppayil S.M. (2011). Evaluation of anti-*Candida* potential of geranium oil constituents against clinical isolates of *Candida albicans* differentially sensitive to fluconazole: Inhibition of growth, dimorphism and sensitization. Mycoses.

[19] Benbelaid F., Khadir A., Abdoine M.A., Bendahou M., Muselli A., Costa J. (2014). Antimicrobial activity of some essential oils against oral multidrug-resistant *Enterococcus faecalis* in both planktonic and biofilm state. Asian Pac. J. Trop. Biomed..

[20] Kotan R., Kordali S., Cakir A. (2007). Screening of antibacterial activities of twenty-one oxygenated monoterpenes. Z. Naturforsch. C.

[21] Inouye S., Takizawa T., Yamaguchi H. (2001). Antibacterial activity of essential oils and their major constituents against respiratory tract pathogens by gaseous contact. J. Antimicrob. Chemother..

[22] Faleiro M.L. (2011). The mode of antibacterial action of essential oils. Science Against Microbial Pathogens: Communicating Current Research and Technological Advances.

[23] Szabó M.A., Varga G.Z., Hohmann J., Schelz Z., Szegedi E., Amaral L., Molnár J. (2010). Inhibition of quorum-sensing signals by essential oils. Phytother. Res..

[24] Sanchez C.J., Mende K., Beckius M.L., Akers K.S., Romano D.R., Wenke J.C., Murray C.K. (2013). Biofilm formation by clinical isolates and the implications in chronic infections. BMC Infect. Dis..

[25] Davies A., Roberts W. (1969). The cell wall of a chlorhexidine-resistant *Pseudomonas*. Biochem. J..

[26] Hassan K.A., Jackson S.M., Penesyan A., Patching S.G., Tetu S.G., Eijkelkamp B.A., Brown M.H., Henderson P.J., Paulsen I.T. (2013). Transcriptomic and biochemical analyses identify a family of chlorhexidine efflux proteins. Proc. Natl. Acad. Sci. USA.

[27] Kulik E.M., Waltimo T., Weiger R., Schweizer I., Lenkeit K., Filipuzzi-Jenny E., Walter C. (2015). Development of resistance of mutans streptococci and *Porphyromonas gingivalis* to chlorhexidine digluconate and amine fluoride/stannous fluoride-containing mouthrinses, *in vitro*. Clin. Oral Investig..

[28] Łysakowska M.E., Ciebiada-Adamiec A., Sienkiewicz M., Sokołowski J., Banaszek K. (2015). The cultivable microbiota of primary and secondary infected root canals, their susceptibility to antibiotics and association with the signs and symptoms of infection. Int. Endod. J..

[29] Syed S.A., Loesche W.J. (1972). Survival of human dental plaque flora in various transport media. Appl. Microbiol..

[30] Kariyama R., Mitsuhata R., Chow J.W., Clewell D.B., Kumon H. (2000). Simple and reliable multiplex PCR assay for surveillance isolates of vancomycin-resistant enterococci. J. Clin. Microbiol..

[31] EUCAST. http://www.eucast.org/clinical_breakpoints/Clinicalbreakpoints-bacteriav2.0.

[32] Clinical and Laboratory Standard Institute (CLSI) (2009). Performance Standards for Antimicrobial Susceptibility Testing: Nineteenth Informational Supplement.

[33] Adams R.P. (2007). Identification of Essential Oil Components by Gas Chromatography/Mass Spectroscopy.

[34] Joulain D., König W.A. (1998). The Atlas of Spectral Data of Sesquiterpene Hydrocarbons.

[35] Andrews J.M. (2001). Determination of minimum inhibitory concentrations. J. Antimicrob. Chemother..

[36] Baca P., Junco P., Arias-Moliz M.T., González-Rodríguez M.P., Ferrer-Luque C.M. (2011). Residual and antimicrobial activity of final irrigation protocols on *Enterococcus faecalis* biofilm in dentin. J. Endod..

